# Mortality from diseases of the circulatory system in Brazil and its relationship with social determinants focusing on vulnerability: an ecological study

**DOI:** 10.1186/s12889-022-14294-3

**Published:** 2022-10-20

**Authors:** Luiz A. V. M. Bastos, Jose L. P. Bichara, Gabriela S. Nascimento, Paolo B. Villela, Glaucia M. M. de Oliveira

**Affiliations:** Federal University of Rio de Janeiro, Rio de Janeiro, Brazil

**Keywords:** Diseases of the circulatory system, Ischemic heart diseases, Social determinants, Municipal human development index, MHDI, Social vulnerability index, SVI

## Abstract

**Background:**

Deaths from diseases of the circulatory system and ischemic heart diseases are declining, but slowly in developing countries, emphasizing its probable relationship with determinants of social vulnerability.

**Objectives:**

To analyze the temporal progression of mortality rates of diseases of the circulatory system and ischemic heart diseases from 1980 to 2019 and the association of the rates with the Municipal Human Development Index and Social Vulnerability Index in Brazil.

**Methods:**

We estimated the crude and standardized mortality rates of diseases of the circulatory system and ischemic heart diseases and analyzed the relationship between the obtained data and the Municipal Human Development Index and Social Vulnerability Index. Data on deaths and population were obtained from the DATASUS. The Municipal Human Development Index and the Social Vulnerability Index of each federative unit were extracted from the websites *Atlas Brazil* and *Atlas of Social Vulnerability*, respectively.

**Results:**

The age-standardized mortality rates of diseases of the circulatory system and ischemic heart diseases showed a downward trend nationwide, which was unequal across the federative units. There was an inversely proportional relationship between the standardized mortality rates of diseases of the circulatory system and ischemic heart diseases and the Municipal Human Development Index. The downward mortality trend was observed when the indices were greater than 0.70 and 0.75, respectively. The Social Vulnerability Index was directly proportional to the standardized mortality rates of diseases of the circulatory system and ischemic heart diseases. An upward mortality trend was observed with a Social Vulnerability Index greater than 0.35.

**Conclusions:**

Social determinants represented by the Municipal Human Development Index and the Social Vulnerability Index were related to mortality from diseases of the circulatory system and ischemic heart diseases across the Brazilian federative units. The units with most development and least social inequalities had the lowest mortality from these causes. The most vulnerable die the most.

**Supplementary Information:**

The online version contains supplementary material available at 10.1186/s12889-022-14294-3.

## Background

Diseases of the circulatory system (DCS) are the leading causes of death worldwide. According to data from the World Health Organization, DCS accounted for more than 15 million deaths in 2019, representing 27% of the deaths worldwide, including more than 75% of those in developing countries [1, 2]. Among the DCS, ischemic heart diseases (IHD) accounted for most deaths, i.e., 8.9 million in 2019 [2]. In Brazil, DCS affected 13,702,303 people in 2017 and have been the leading cause of death since 1960. According to estimates from the Global Burden of Disease (GBD), DCS accounted for 388,268 deaths in Brazil in 2017, representing 27.3% of the total deaths in the country [3]. Most deaths, according to the 2017 GBD data, were due to IHD, which accounted for 175,791 (30%) of the deaths [3].

Despite the high prevalence of DCS and IHD, deaths from these diseases have been declining in several countries since the second half of the twentieth century. This phenomenon is explained by improvements in prevention and treatment measures, marked by decreased smoking, improved control of blood pressure and dyslipidemia, and developments in thrombolysis and revascularization [4]. However, a global analysis shows that these diseases decline more slowly in developing countries [5], probably due to socioeconomic factors. International studies have observed this probable association with socioeconomic factors through a comparative analysis between populations with different levels of education [6, 7], ethnicity [8], and income. Brazilian studies have reached similar conclusions comparing the different geographic regions of the country, which have their own inequalities [9–12], while considering socioeconomic factors [13–15].

One way to analyze the socioeconomic determinants and their relationship with mortality from DCS and IHD is using indicators. The Municipal Human Development Index (MHDI) is the most used, for example, a 2018 Brazilian study observed an inverse association between this index and DCS, hypertensive diseases, and cerebrovascular diseases between 2004 and 2013 [14]. The Social Vulnerability Index (SVI) addresses data related to social exclusion and vulnerability and is less known. SVI has been negatively associated with mortality from cerebrovascular disease in a 2021 Brazilian study, but studies associating vulnerability with DCS and IHD do not exist, what makes our work unique and innovative [15].

Thus, it is becoming increasingly necessary to address the influence of regional socioeconomic factors on public health and development of DCS and IHD, considering that the regional social and economic development is accompanied by improved quality of life and health in the population. Based on these considerations, the aim of this study was to analyze the temporal progression of mortality rates of DCS and IHD by sex, age group, federative unit, and geographical region in Brazil from 1980 to 2019, and the relationships between these rates with MHDI and SVI focusing on vulnerability.

## Methods

Ecological study of a time series of deaths due to DCS and IHD that occurred in Brazil between 1980 and 2019 across all age groups and in both sexes, categorized by federative unit and geographic region.

Data on the underlying causes of death were obtained from the Information System on Mortality (*Sistema de Informações sobre Mortalidade*, SIM) website maintained by the Information Technology Department of the Brazilian Unified Health System (*Departamento de Informática do Sistema Único de Saúde*, DATASUS) of the Brazilian Ministry of Health [16]. The data were downloaded into a spreadsheet, and the original files (in CSV format) were converted into XLS format using Excel 2016 (Microsoft Corporation, Seattle, WA, USA) [17], which was also used for data analysis and construction of graphs and tables. The deaths were classified according to the following groups of causes: “Diseases of the Circulatory System” (ICD-9 Chapter 7 [18] and ICD-10 Chapter 9 [19]) and “ischemic heart diseases” (same group name, ICD-9 and ICD-10) [18, 19]. We used ICD-9 codes [18] for deaths occurring between 1980 and 1995 and ICD-10 codes [19] for those occurring between 1996 and 2019.

Information on the resident population was also obtained from the DATASUS website [16], which in turn considered census data from the Brazilian Institute of Geography and Statistics (*Instituto Brasileiro de Geografia e Estatística*, IBGE) from 1980, 1991, 2000, and 2010, intercensal projections up to 2012, and populational projections from 2013 onwards.

We used the direct method to estimate the crude and standardized gross annual mortality rates of DCS and IHD and their rates across sex, age group, and federative unit per 100,000 inhabitants. The age structure of the Brazilian population in the year 2000 was used as the standard.

The MHDI of each federative unit, obtained from the website *Atlas Brasil* [20], derives from the Human Development Index (HDI), and is adapted to municipal and state levels. The MHDI takes into account progress on the basic dimensions of health, education, and income, assessing wealth, literacy, life expectancy, and birth rates. This index ranges from 0 to 1, with numbers closer to 1, indicating greater human development [21].

The SVI is complementary to the MHDI and allows for a unique mapping of exclusion and social vulnerability in the 5565 Brazilian municipalities. The SVI, which synthesizes data on urban infrastructure, human capital, and income/labor, evaluated from sixteen sub-indicators with different weights, indicates the access, absence, or insufficiency of some “assets” in areas of the Brazilian territory, which should, in principle, be available to every citizen [22]. The SVI deals with social discrimination and exclusion and varies from 0 to 1, where 0 is the ideal or perfect situation, and one is the worst. The higher the index, the greater the social vulnerability, therefore, values between 0 and 0,2 represent very low social vulnerability; 0,201 and 0,3: low; 0,301 and 0,4: average; 0,401 and 0,5: high and 0,501 and 1: very high. The SVI of each federative unit was extracted from the website Atlas of Social Vulnerability and is built from indicators from the Atlas of Human Development [23].

We evaluated the relationship between the MHDI categorized by federative unit and the standardized mortality rates from DCS and IHD. First, we analyzed the relationship between the 1991, 2000, and 2010 MHDI and the standardized mortality rate for 2019 based on previous studies with a time lag of approximately 10 years [13]. Then, we evaluated the relationship between the 1991, 2000, and 2010 MHDI and the variation in the standardized mortality rates between 1980 and 2019. Finally, we analyzed the relationship between the MHDI variation between 1991 and 2010 and the variation in the standardized mortality rates between 1980 and 2019.

We also analyzed the relationship between the SVI and the mortality rates of DCS and IHD. We started by evaluating the relationship between the 2000 and 2010 SVI and the standardized mortality rate for the year 2019 based on a time lag of study with MHDI [13] in the absence of SVI studies and, after that, between the 2000 and 2010 SVI and the variation in mortality between 1980 and 2019. Finally, we analyzed the relationship between the SVI variation from 1991 to 2010 and the variation in mortality rates between 1980 and 2019.

For data analysis and construction of tables and graphs, we also used Excel 2016 [17].

## Results

A total of 10,836,004 deaths from DCS and 3,264,828 from IHD were recorded in Brazil between 1980 and 2019. Regarding IHD deaths across the country’s geographic regions, 1,781,663 (54.6%) occurred in the Southeast, followed by 607,277 (18,6%) in the Northeast, 604,479 (18.5%) in the South, 165,879 (5.1%) in the Midwest, and 105,530 (3.2%) in the North.

The age-standardized mortality rates of DCS and IHD in both sexes showed a downward trend nationwide during the period, from 233.26 to 111.58 per 100,000 inhabitants for DCS and 65.15 to 36.16 per 100,000 inhabitants for IHD, a decrease of about 52.1 and 44.5%, respectively.

**Fig. 1:**
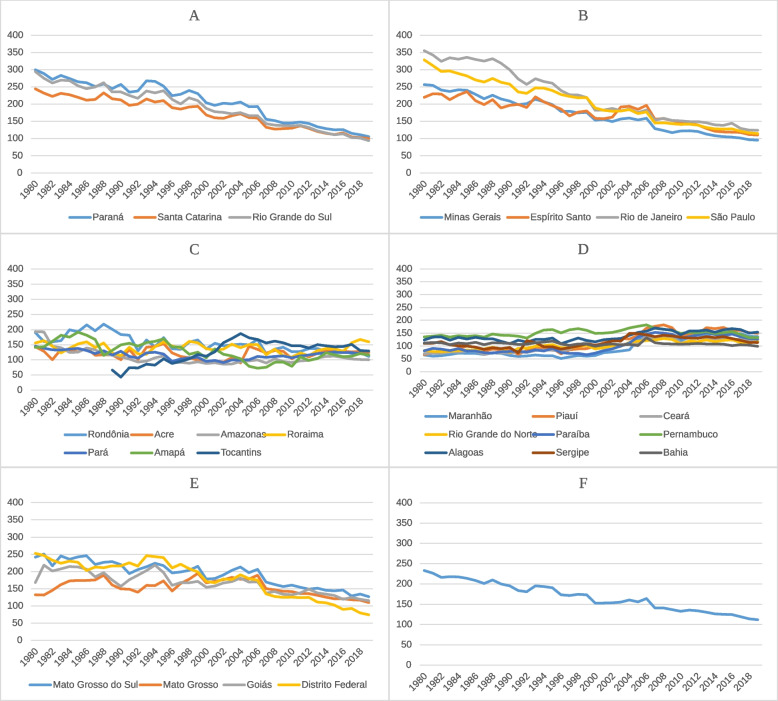
DCS standardized mortality rate, by FedU, region, and national from 1980 to 2019. Variations in age-standardized mortality rates of Diseases of the Circulatory System (DCS) per 100,000 inhabitants in both sexes and categorized by Federative Unit (FedU) in the South (**A**), Southeast (**B**), North (**C**), Northeast (**D**), and Midwest (**E**) regions of Brazil and the combined national rate (**F**) between 1980 and 2019

**Fig. 2:**
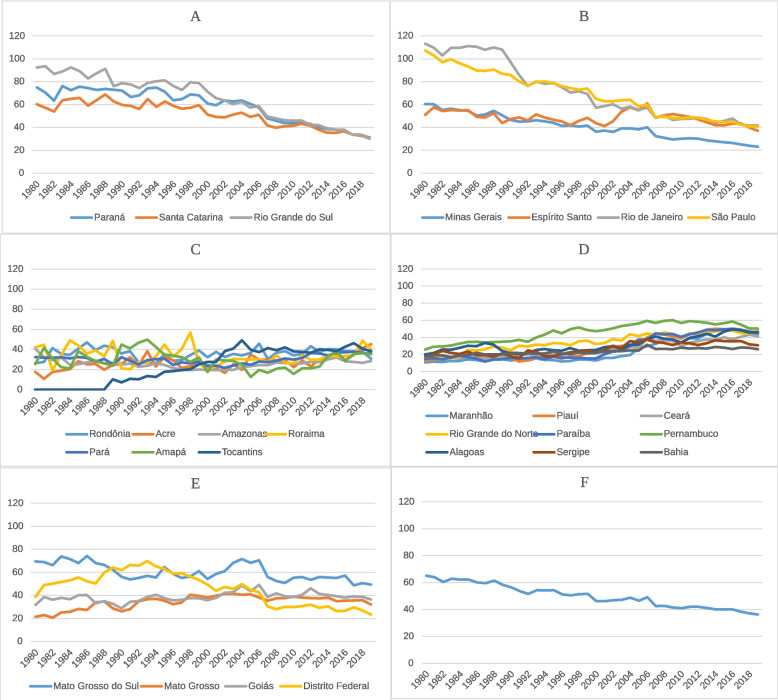
IHD standardized mortality rate, by FedU, region, and national from 1980 to 2019. Variations in age-standardized mortality rates of Ischemic Heart Diseases (IHD) 100,000 inhabitants in both sexes and categorized by Federative Unit (FedU) in the South (**A**), Southeast (**B**), North (**C**), Northeast (**D**), and Midwest (**E**) regions of Brazil and the combined national rate (**F**) between 1980 and 2019

This trend was not uniform across all geographic regions. The South and Southeast regions showed a relevant decrease in age-standardized mortality rates of DCS and IHD. However, the North and the Midwest showed stable rates, while the Northeast showed an upward trend. This analysis is shown in the Figures below, which represent the variation in age-standardized mortality rates per 100,000 inhabitants in both sexes, by federative unit, divided across the five geographic regions, as well as combined data from the national territory for DCS (Fig. 1) and IHD (Fig. 2).

**Fig. 3:**
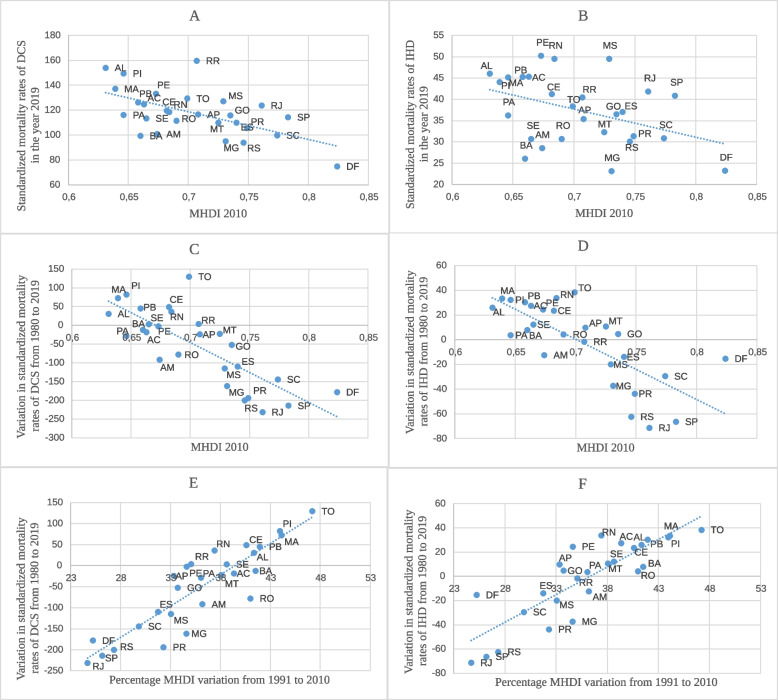
Relationship between DCS and IHD standardized mortality rates and the MHDI from 1991 to 2010. The graphs show the relationship between (**A**) the Federative Units MHDI in 2010 and standardized mortality rates of Diseases of the Circulatory System (DCS) and Ischemic Heart Diseases (IHD) in the year 2019; (**B**) the Federative Units MHDI in 2010 and the variation in standardized mortality rates of (**C**) DCS and (**D**) IHD from 1980 to 2019; and the variation in standardized mortality rates of (**E**) DCS and (**F**) IHD from1980 to 2019 and the percentage MHDI variation from 1991 to 2010

Figure 3 shows the relationship between the standardized mortality rate of DCS and IHD and the MHDI. Figure 3A and B show an inversely proportional relationship between the MHDI of the federative units in 2010 and the standardized mortality rate of DCS and IHD in the year 2019, indicating that the higher the number of deaths, the lower the MHDI of the federative unit. As indicated in Fig. 3C and D, the lower the MHDI of the federative unit in 2010, the greater the increase in standardized mortality rates of DCS and IHD. There was a downward trend when the indices were greater than 0.70 and 0.75, respectively, while the relationship with the MHDI was maintained, with the greatest reduction observed in the federative units with the highest index. Figure 3E and F show the relationship between the variation in the standardized mortality rates of DCS and IHD between 1980 and 2019 and the percentage MHDI variation between 1991 and 2010. Notably, the federative units with the least MHDI variation in the period showed decreasing mortality, indicating that a high absolute MHDI is probably more important than a progressive improvement in this index. The Pearson correlation coefficient of the MHDI with DCS and IHD was 0.89 and 0.84, respectively.

**Fig. 4:**
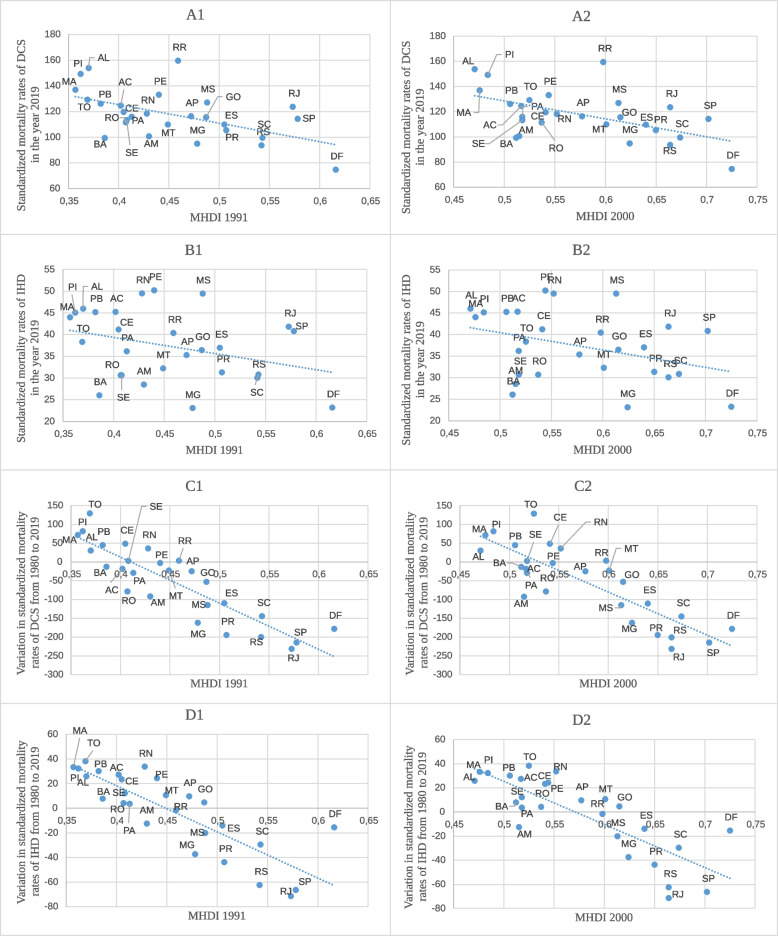
Relationship between DCS and IHD standardized mortality rates of and MHDI in 1991 and 2000. The graphs show the relationship between (A1/A2) the Federative Units MHDI in 1991 and 2000, respectively, and standardized mortality rates of DCS and IHD in the year 2019; (B1/B2) the Federative Units MHDI in 1991 and 2000, respectively, and the variation in standardized mortality rates of (C1/C2) DCS and (D1/D2) IHD from 1980 to 2019

Figure 4 shows the relationship between the standardized mortality rate of DCS and IHD and the MHDI for the previous years 1991 and 2000. Figures 4A1/A2 and 4B1/B2 show an inversely proportional relationship between the MHDI of the federative units in 1991 and 2000 and the standardized mortality rate of DCS and IHD in the year 2019, indicating that the higher the number of deaths, the lower the MHDI of the federative unit as had already been seen in relation to the year 2010. As indicated in Figures 4C1/C2 and 4D1/D2, the lower the MHDI of the federative unit for the previous years 1991 and 2000, the greater the increase in standardized mortality rates of DCS and IHD. There was a downward trend when the indices were greater than 0.70 and 0.75, respectively, while the relationship with the MHDI was maintained, with the greatest reduction observed in the federative units with the highest index as had already been seen in relation to the year 2010.

**Fig. 5:**
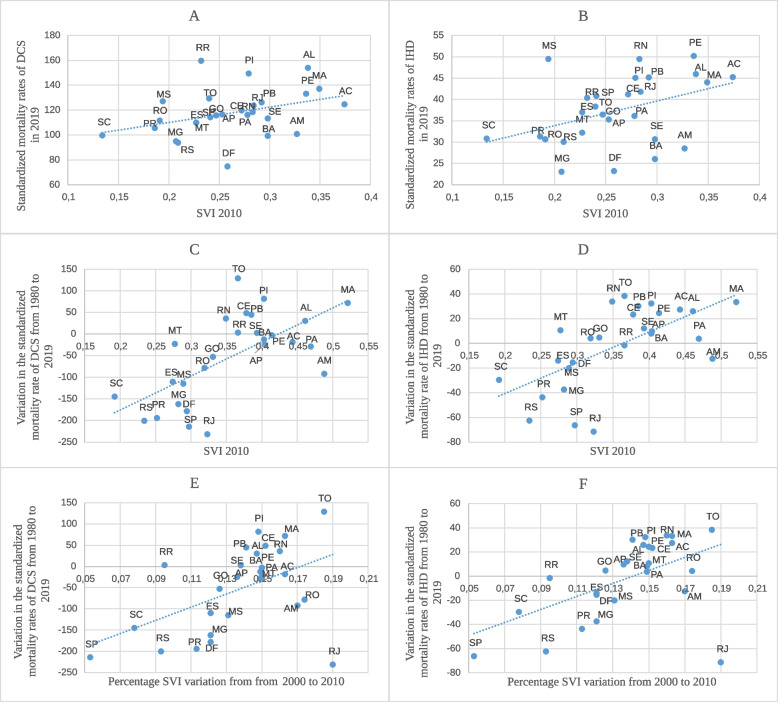
Relationship between DCS and IHD standardized mortality rates and the SVI from 2000 to 2010. The graphs show the relationship between the Federative Units Social Vulnerability Index (SVI) in 2010 and the standardized mortality rates of (**A**) Diseases of the Circulatory System (DCS) and (**B**) Ischemic heart Diseases (IHD) in 2019; between the Federative Units SVI in 2010 and the variation in the standardized mortality rate of (**C**) DCS and (**D**) IHD from 1980 to 2019; and between the variation in the standardized mortality rates of (**E**) DCS and (**F**) IHD from 1980 to 2019 and the percentage SVI variation from 2000 to 2010

Figure 5 shows the relationship between the SVI and the standardized mortality rates of DCS and IHD. Figure 5A and B show a directly proportional relationship between the SVI of the federative units in 2010 and the standardized mortality rate of DCS and IHD in the year 2019. As indicated, the lower the SVI, the lower the mortality rate. As shown in Fig. 5C and D, the higher the federative unit SVI in 2010, the greater the increase in the standardized mortality rate of DCS and IHD between 1980 and 2019. There was an upward trend when the index was greater than 0.35 while maintaining the directly proportional relationship with the SVI, with a greater reduction in the federative units with the lowest indices, particularly when the index was below 0.35. Figure 5E and F show the relationship between the variation in the standardized mortality rates of DCS and IHD between 1980 and 2019 and the variation in the SVI between 2000 and 2010. Notably, the federative units with the least SVI variation in the period showed decreasing mortality, indicating that a good absolute SVI is probably more important than a progressive improvement of this index, as observed with the MHDI. The Pearson correlation coefficient of the SVI with DCS and IHD was 0.49 and 0.53, respectively.

**Fig. 6:**
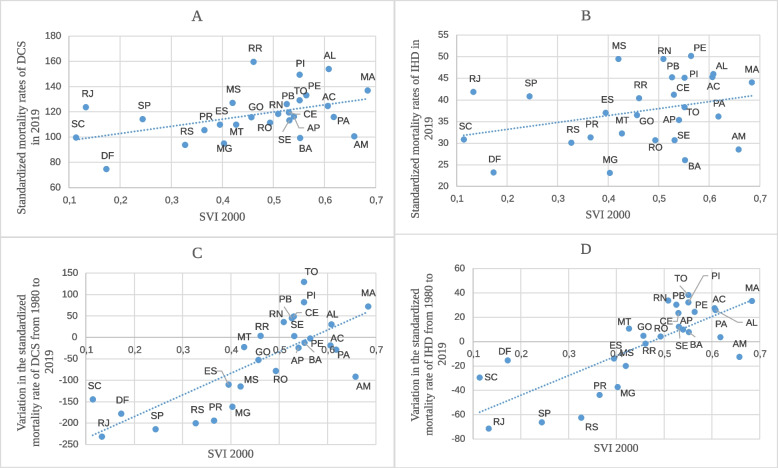
Relationship between DCS and IHD standardized mortality rates and the SVI from 2000. The graphs show the relationship between the Federative Units Social Vulnerability Index (SVI) in 2000 and the standardized mortality rates of (**A**) Diseases of the Circulatory System (DCS) and (**B**) Ischemic heart Diseases (IHD) in 2019 and between the Federative Units SVI in 2000 and the variation in the standardized mortality rate of (**C**) DCS and (**D**) IHD from 1980 to 2019

Figure 6 shows the relationship between the SVI and the standardized mortality rates of DCS and IHD. Figure 6A and B show a directly proportional relationship between the SVI of the federative units in 2000 and the standardized mortality rate of DCS and IHD in the year 2019. As indicated, the lower the SVI, the lower the mortality rate as had already been seen in relation to the year 2010. As shown in Fig. 6C and D, the higher the federative unit SVI in 2000, the greater the increase in the standardized mortality rate of DCS and IHD between 1980 and 2019. There was an upward trend when the index was greater than 0.35 while maintaining the directly proportional relationship with the SVI, with a greater reduction in the federative units with the lowest indices, particularly when the index was below 0.35 as had already been seen in relation to the year 2010.

## Discussion

The present study showed an inverse relationship between the MHDI and the standardized mortality rates of DCS and IHD of the Brazilian Federal Units, so the highest MHDI showed the more pronounced degrees in mortality rates. In addition to a direct relationship with the SVI, because the lower the SVI, the greater the drop in mortality. Importantly, improvements in indicators do not necessarily reflect improvements in mortality rates unless absolute values of 0,7 (MHDI) or 0,35 (SVI) had been reached, as noted in Figs. 3 and 5.

In cases of TO, MA, AC and AM for example, despite most significant improvements in MHDI (Fig. 3) and SVI (Fig. 5) between 2000 and 2010, mortality rates also reached worse values. In contrast, in RJ, DF, SC and SP, which showed less variations in indicators in the same period and reached minimum values of 0,7 for MHDI or 0,35 for SVI, had best improvements in mortality rates, reinforcing that absolute value of the index has probably more impact in mortality reduction than its variation along the time.

Moreover, we observed a high prevalence of DCS and IHD, with 10,836,004 deaths from DCS and 3,264,828 deaths from IHD in Brazil between 1980 and 2019, and a downward trend in mortality rates of DCS and IHD over the period. However, this decrease was uneven across the country’s federative units and geographic regions. It was more prominent in the South and Southeast regions, which have more excellent socio-economic development, and the Northeast region. At the same time, the rates remained stable in the North and Midwest regions, with the last three areas being the poorest and most vulnerable in the country. A previous study with data from the GBD 2015 had already observed this trend of more pronounced reduction in mortality from cardiovascular diseases in the South and Southeast regions, which concentrate the most significant financial gain in the country, compared to the North, Northeast, and Midwest regions which have the highest vulnerability and social inequality [10].

This observation is consistent with reports from previous studies, including those pointing out an uneven decrease in the number of deaths from DCS between 1990 and 2015 across the country’s geographic regions, more pronounced in states in the South and Southeast regions and less pronounced in the North and Northeast regions [10–12]. However, these studies did not evaluate the relationship between the differences in mortality trends and social determinants while only reporting the decreasing mortality as less prominent in regions with greater development. Other studies went further, correlating socioeconomic factors – such as education level and income – with hypertension rates and reporting an inverse correlation between both. The likely justification for this observation is that the higher the education level of an individual, the better is his or her understanding of health information and recommendations, with consequent greater adherence to treatment in terms of use of medications, changes in lifestyle and eating habits, and prevention of risk factors [24–26]. Conversely, a low income also influences treatment adherence, as it interferes with optimal access to medications, healthy diet, and physical activity [25].

A study evaluating the association between mortality from DCS in municipalities of the state of Rio de Janeiro from 1979 to 2010 and the Gross Domestic Product (GDP) per capita obtained from the Institute of Applied Economic Research (*Instituto de Pesquisa Econômica Aplicada*, IPEA) showed a decrease in mortality from DCS associated with a GDP increase with a time lag of more than 10 years [13]. In 2018, another study evaluating the association of DCS, hypertensive diseases, and cerebrovascular diseases with the HDI between the years 2004 and 2013 showed a significant inverse association between socioeconomic factors and mortality from these diseases [14].

Our study went even further by carrying out this analysis over a longer period – from 1980 to 2019 – and comparing socioeconomic factors with DCS and IHD mortality rates focusing on vulnerability. To accomplish this, we used two different social determinants in our analysis. The first was the MHDI, which is more commonly used and previously applied in other studies incorporating assessments of health, education, and income with an interval between the index and the result of more than 10 years. The second was the SVI, which is a lesser-known and makes our analysis unique when applying this index that has not been used in previous studies on DCS and IHD mortality rates, expanding analysis to vulnerability. Given the absence of studies with this indicator, there is no data in the literature on the time required between the change in the index and its influence in DCS and IHD.

Naturally, we must keep in mind the genetic influences associated with the development of DCS and IHD, in addition to lifestyle habits associated with risk factors such as a diet rich in salt and fat, obesity, sedentary lifestyle, alcohol consumption, and smoking. The initial step toward improving the high incidence rates of cardiovascular disease in Brazil is to invest in human development across different regions of the country and reduce social vulnerability to allow for the fulfillment of the constitutional rights of each citizen, including, for example, access to education and awareness of the possible causes of the diseases addressed in this study, access to food appropriate to the individual’s nutritional requirements, quality housing and health, as well as access to medications, prophylactic methods, and adequate medical treatments.

In short, in view of the relationship observed in this study between the HDI and the frequency of DCS and IHD in the population, it is important to emphasize the importance of government investment in the social and economic development of the country’s microregions and the nation as a whole as a way of maintaining public health.

Limitations of this study include its observational design, which does not allow for a causality conclusion but raises hypotheses and awareness that can help implement necessary political, social, and administrative measures. The presented data demonstrate that improved mortality results from DCS accompany the progression of the social development indices analyzed in the study. Another relevant limitation of this study is that the information was retrieved from a database, with possible biases generated by data entry errors like deaths attributed to ill-defined causes, underreporting, and garbage codes [27]. This is aggravated by the fact that regions such as the North and Northeast of the country always had more garbage codes and underreporting with a later and significant improvement in the quality of this data collection [27]. Finally, another limitation is the possibility of an ecological bias as mortality is assessed at an individual level, but social determinants are being measured at the group level; but is an issue inherent to the theme because, when you are analyzing social determinants, you work on the community spectrum. This is even clearer when we think of vulnerability as this indicator, which deals with the failure of a given community to meet basic needs, with no individual data available on this theme. Another limitation is that the time required for a change in the MHDI or SVI to influence mortality from DCS or IHD is not yet fully established, especially when it comes to IVS, due to a lack of studies in the area.

Future perspectives: Our work reaches its object when evaluating the relationship between socioeconomic factors, focusing on social vulnerability and mortality due to DCS and IHD. However, other factors influence mortality, such as the health system, risk factors beyond the death registry system itself, which can be affected by the diagnostic method, diagnostic criteria, or even the choice of the technique for a fundamental cause that underestimates the influence of chronic diseases on the final result. Future studies evaluating multiple causes or comparing the technological distribution of diagnostic material with vulnerability will be necessary to clarify the theme better [28, 29].

## Conclusions

This study shows a national downward trend in mortality from DCS and IHD across the federative units of Brazil. However, the trend was unequal across the geographic regions, probably due to differences in social determinants, represented by the MHDI and the SVI. The regions with the most development and least social inequalities presented the lowest mortality from these causes. The most vulnerable die the most.

## Supplementary Information

**Additional file 1.**

## Data Availability

All data generated or analysed during this study are included in this published article [and its supplementary information files.

## Abbreviations

DATASUS: Departamento de Informática do Sistema Único de Saúde
DCS: Diseases of the circulatory system
GDP: Gross Domestic Product
GBD: Global Burden of Disease
IBGE: Instituto Brasileiro de Geografia e Estatística
IHD: ischemic heart diseases
IPEA: Instituto de Pesquisa Econômica Aplicada
MHDI: Municipal Human Development Index
SVI: Social Vulnerability Index

## References

[1] World Health Organization - WHO (2021). Newsroom. Fact Sheets. Detail. Cardiovascular Disease.

[2] World Health Organization - WHO (2020). The top 10 causes of death.

[3] GMM O, LCC B, Polanczyk CA, Biolo A, Nascimento BR, Malta DC (2020). Cardiovascular Statistics – Brazil 2020. Sociedade Brasileira de Cardiologia.

[4] Mensah GA, Wei GS, Sorlie PD, Fine LJ, Rosenberg Y, Kaufmann PG (2017). Decline in cardiovascular mortality: possible causes and implications. Circ Res.

[5] MORAN AE, Forouzanfar MH, Roth GA, Mensah GA, Ezzati M, Murray CJL (2014). Temporal trends in ischemic heart disease mortality in 21 World regions, 1980 to 2010 the global burden of disease 2010 study. Circulation.

[6] Holme I, Helgeland A, Hjermann I, Leren P, Lund-Larssen PG (1977). Coronary risk factors and socioeconomic status – the Oslo study. Lancet.

[7] Abbasi SH, De Leon AP, Kassain SE, Karimi A, Sundin O, Jalali A (2015). Socioeconomic status and in-hospital mortality of acute coronary syndrome: can education and occupation serve as preventive measures?. Int J Prev Med.

[8] Nadruz W, Claggett B, Henglin M, Shah AM, Skali H, Rosamond WD (2018). Widening racial differences in risks for coronary heart disease. Circulation.

[9] Astrom DO, Sundquist J, Sundquist K (2018). Differences in declining mortality rates due to coronary heart disease by neighborhood deprivation. J epidemiol. Community Dent Health.

[10] LCC B, Nascimento BR, VMA P, Duncan BB, IJM B, Malta DC (2017). Variations and particularities in cardiovascular disease mortality in Brazil and Brazilian states in 1990 and 2015: estimates from the global burden of disease. Rev Bras Epidemiol.

[11] Mansur AP, Favarato D (2016). Mortality due to cardiovascular diseases in women and men in the five Brazilian regions, 1980-2012. Arq Bras Cardiol.

[12] Baena CP, Chowdhury R, Schio NA, Sabbag AE, Guarita-Souza LC, Olandoski M (2013). Ischaemic heart disease deaths in Brazil: current trends, regional disparities and future projections. Heart.

[13] Soares GP, Klein CH, Silva NAS, Oliveira GMM (2018). Evolution of mortality from diseases of the circulatory system and of gross domestic product per capita in the Rio de Janeiro state municipalities. Int J Cardiovasc Sci.

[14] Villela PB, Klein CH, GMM O (2019). Socioeconomic factors and mortality due to cerebrovascular and hypertensive disease in Brazil. Rev Port Cardiol (Engl Ed).

[15] CDF S, Oliveira DJ, Silva LF, Santos CD, Pereira MC, JPS P (2021). Cerebrovascular disease mortality trend in Brazil (1996 to 2015) and association with human development index and social vulnerability. Arq Bras Cardiol.

[16] Brazil. Ministry of Health. Executive Secretariat. Datasus. Health Information. Vital statistics. Available at: http://siab.datasus.gov.br/DATASUS/index.php?area=0205 . Accessed in 6 April 2022.

[17] Microsoft Excel (2020). Microsoft corporation. Version.

[18] World Health Organization (WHO). Manual of the international classification of diseases, injuries and causes of death, 9th. rev. São Paulo; 1978. Available at: Manual of the international statistical classification of diseases, injuries, and causes of death : based on the recommendations of the ninth revision conference, 1975, and adopted by the Twenty-ninth World Health Assembly (who.int). Accessed 6 April 2022.

[19] World Health Organization (WHO). International statistical classification of diseases and related health problems: international classification of diseases. 10th rev. São Paulo: EDUSP; 1995. Available at: ICD-10 : international statistical classification of diseases and related health problems : tenth revision (who.int). Accessed 6 April 2022.

[20] Atlas of Human Development in Brazil. Available at: http://www.atlasbrasil.org.br/. Accessed 6 April 2022.

[21] Institute of Applied Economic Research– Available at: O que é? IDH (ipea.gov.br). Acessed 6 April 2022.

[22] Economic observatory de Contagem. Institute of Applied Economic Research,. Available at: http://www.contagem.mg.gov.br/observatorio/ivs/. Acessed 6 April 2022.

[23] Institute of Applied Economic Research. Available at: https://ipea.gov.br/portal/index.php?option=com_content&id=26073:ipea-mapeia-vulnerabilidade-social-nos-municipios-brasileiros&directory=1#:~:text=O%20Atlas%20da%20Vulnerabilidade%20Social%20nos%20Munic%C3%ADpios%20Brasileiros,orienta%C3%A7%C3%A3o%20de%20gestores%20p%C3%BAblicos%20municipais%2C%20estaduais%20e%20federais. Acessed in 6 April 2022.

[24] Pessuto J, de Carvalho EC. Risk factors to patients with arterial hypertension (scielo.br). Rev Latino-Am Enfermagem. 1998;6(1). 10.1590/S0104-11691998000100006. 10.1590/s0104-11691998000100006 9592550

[25] Pires CGS, Mussi FC. Health beliefs for the control of arterial hypertension (scielo.br). Ciênc saúde coletiva. 2008;13(suppl 2). 10.1590/S1413-81232008000900030. 10.1590/s1413-81232008000900030 19039409

[26] da Costa JSD, Barcelos FC, Sclowitz ML, IKT S, Castanheira M, MTA O, et al. Hypertension prevalence and its associated risk factors in adults: a population-based study in Pelotas. Arq Bras Cardiol. 2007;88(1). 10.1590/S0066-782X2007000100010. 10.1590/s0066-782x2007000100010 17364120

[27] FERREIRA LCM, Nogueira MC, Carvalho MS, Teixeira MTB. (2020). Mortality due to acute myocardial infarction in Brazil from 1996 to 2016: 21 years of disparities in Brazilian regions - ABC Cardiol. Arq Bras Cardiol.

[28] Piffaretti C, Moreno-Betancur M, Lamarche-Vadel A, Rey G (2016). Quantifying cause-related mortality by weighting multiple causes of death. Bull World Health Organ.

[29] Timonin S, Shkolnikov VM, Andreev E, Magnus P, Leon DA (2022). Evidence of large systematic differences between countries in assigning ischaemic heart disease deaths to myocardial infarction: the contrasting examples of Russia and Norway. Int J Epidemiol.

